# Response to Dr. Masaya Miyauchi and Dr. Teruhiko Imamura, Clinical implication of COVID‐19 associated bradycardia

**DOI:** 10.1002/clc.23675

**Published:** 2021-07-09

**Authors:** Sabina Kumar, Christina Arcuri, Sumanta Chaudhuri, Rahul Gupta, Mahendra Aseri, Pranav Barve, Shivang Shah

**Affiliations:** ^1^ Department of Internal Medicine Hemet Global Medical Center Hemet California USA; ^2^ Division of Cardiology Loma Linda University School of Medicine Loma Linda California USA; ^3^ Division of Cardiology University of California Riverside School of Medicine Riverside California USA

To the Editor,

Thank you Dr. Imamura and Colleagues. We appreciate your input and thoughts on further studying bradycardia and COVID‐19. Our large‐scale multicenter retrospective study included over 1000 patients with COVID‐19, and found that absolute bradycardia was noted in 24.9% of the study cohort and these individuals were found to have a significant increase in mortality.

We agree with you that differentiating the type of bradycardia in a patient is important to determine the etiology, and ultimately, the appropriate treatment. However, the data included in our study was obtained from March, 2020 to August, 2020 during the early phase of the pandemic when staff exposure to COVID patients was limited. As a result, echocardiogram and 12‐lead EKG's were typically completed only once during admission, if indicated. As this was a retrospective study, we had to examine whatever data and tests had been collected. We believe that in the future a prospective study evaluating the etiology of bradycardia with these tests is important to obtain thorough diagnosis and treatment.^1^

Given the high prevalence of absolute bradycardia in our study population, clinicians should carefully consider next steps in treating absolute bradycardia in the inpatient setting. The European Society of Cardiology suggested an initial trial of isoprenaline and atropine, and if bradycardia is sustained, temporary pacing should be considered.^2^ However, a recent Italian paper proposed an early permanent pacemaker implantation to avoid the increased risk of infection that exists when a temporary pacemaker is placed.^3^ Brignole et al reported that a temporary pacemaker before a permanent pacemaker implantation is 2.5 times more likely to develop a risk of infection versus the latter.^4^ Chintz et al had seven patients with COVID‐19 that developed bradyarrhythmias in which they placed three temporary and four leadless pacemakers. Despite placing the pacemakers, they noted a short term morbidity and subsequent death due to COVID‐19 complications in five out of seven patients.^5^ Based on the current literature available and the novel analysis in our study, it is important that that the international community research the next steps to address bradycardia and COVID‐19.

A future study, specifically a retrospective cohort separating pre‐existing heart failure from those without pre‐existing heart failure may help to differentiate respiratory failure versus heart failure as the cause of mortality. As for myocarditis in COVID‐19; The CDC has recently reported that myocarditis post mRNA COVID‐19 vaccination is a well‐known complication.

We look forward to any further input and suggestions that Dr. Imamura and Colleagues may have.

## CONFLICT OF INTEREST

The author has declared no conflicts of interest for this article.

## Data Availability

The data that support the findings of this study are available from the corresponding author upon reasonable request.
